# Sanitary Care of Enlisted Men

**Published:** 1918-11

**Authors:** Antoinette Funk


### SANITARY CARE OF ENLISTED MEN

BY ANTOINETTE FUNK

When a man is selected for military service the immediate anxiety, the immediate concern, is his destination, his housing, feeding, clothing and health.

The new soldier is under the direction of the Provost Marshal General's department from the time he is accepted until he takes train for the camp he is assigned to. Then the transportation department takes him in charge. If his journey is a long one he travels by Pullman or tourist sleeper. Meals are provided to him along the way, at a maximum cost of sixty cents by the government.

Under a recent ruling the selected man is immediately given an armband. This is an insignia of military standing and is worn until he is fitted with a uniform. This arm-band carries the same authority, protection and responsibility that the uniform does. The enemy would have a right to fire upon him or take him prisoner, and anyone selling him liquor would be subject to prosecution under the federal law.

Arriving at his cantonment the soldier is assigned to quarters, usually in a two-story wooden building, with plenty of air and sun-light, and with the cleanest of floors

—floors that would meet the old-time test "clean enough to eat from." He sleeps in a well ventilated room with other soldiers, but not too many, the number being regulated by the cubic feet of air space in the chamber. The army bed is an extra width cot with good steel springs and bedding suited to the weather and climate; never less than two blankets are assigned him, all wool blankets, khaki color. Sometimes he gets three and two thick comforters more if weather demands.

Lavatories are located at the rear of these quarters, with water pressure and fixtures of a design similar to that used in the best hotels in the country, and for every company unit there are from four to six shower baths. Cleanliness of person and surroundings are absolute requirements of the United States army. Every possible precaution is taken by the sanitary corps to insure that the camp conditions are 100 per cent. sanitary.

Drainage is installed along strictly scientific lines, and the most scientific disposition is made of all camp sewage. During previous wars more men have died from preventable disease than from bullet wounds. During the civil war soldiers perished by thousands from typhoid, camp fever, dysentery and kindred diseases resulting from unsanitary conditions about the camp. Those days are gone. Surgeon General Gorgas, who made the building of the Panama Canal possible by draining the Canal Zone and fitting it for human habitation, is in charge of the army sanitation.

As soon as the soldier is assigned to quarters he is given the most searching physical examination. All scientific medical tests are applied to detect disease. For instance, if there are indications of tubercular infection the patient is put under observation that there may be no mistake in the diagnosis. If there is incipient trouble he is sent to one of the army sanitariums and restored to health. If his case is advanced he is relieved from military service or exempted until physically fit.

Besides the examining surgeon there is the dentist. Teeth are put in good condition here, and there are dentists overseas to keep them in good condition. Also there is an orthopedic surgeon to examine the soldier's feet. It has been said that during past wars there were more desertions from foot trouble than all other causes combined. The attention given to the selection of shoes for the soldiers in the American army is a sidelight on the care we give our fighting men.

When a soldier gets his first pair of shoes he gets a pair that fit his feet. No account is taken of the size he wore before. His feet are placed in a cunningly devised form where the length and width are exactly determined. He bears his weight on this little machine and an officer and a non-commissioned officer take the size record of both feet, his name, company and regiment. Then he puts on a pair of shoes of the size called for. But that doesn't end it. There is a further device that checks on the measuring machine and catches any human error in recording. This is put inside his shoe and he runs down an incline of 30 degrees, striking his heels on the cleats nailed to it. If this little machine does not make itself felt and the shoe after examination by an officer is found to be satisfactory the man is fitted and his size is added to his service record.

Our soldiers are provided with clean socks, and at the end of long marches the feet are carefully inspected by the surgeon in charge.

No army in the world has ever attained such a health record as ours, the death rate being eight out of every thousand, here and abroad. This would be even lower but for the large number of men who come down with diseases to which they were exposed before leaving home.

The average gain in weight of the American soldiers since entering the service is twelve pounds per man.

